# Diabetes-Related Distress and Depressive Symptoms Are Not Merely Negative over a 3-Year Period in Malaysian Adults with Type 2 Diabetes Mellitus Receiving Regular Primary Diabetes Care

**DOI:** 10.3389/fpsyg.2017.01834

**Published:** 2017-10-17

**Authors:** Boon-How Chew, Rimke C. Vos, Rebecca K. Stellato, Guy E. H. M. Rutten

**Affiliations:** ^1^Department of Family Medicine, Faculty of Medicine and Health Sciences, Universiti Putra Malaysia, Serdang, Malaysia; ^2^Department of General Practice, Julius Center for Health Sciences and Primary Care, University Medical Center Utrecht, Utrecht, Netherlands

**Keywords:** diabetes distress, depression, type 2 diabetes mellitus, quality of life, complications, HbA1c, primary care, NCT02730754, https://clinicaltrials.gov/ct2/show/NCT02730754.

## Abstract

For people with type 2 diabetes mellitus (T2DM) the daily maintenance of physical and psychological health is challenging. However, the interrelatedness of these two health domains, and of diabetes-related distress (DRD) and depressive symptoms, in the Asian population is still poorly understood. DRD and depressive symptoms have important but distinct influences on diabetes self-care and disease control. Furthermore, the question of whether changes in DRD or depressive symptoms follow a more or less natural course or depend on disease and therapy-related factors is yet to be answered. The aim of this study was to identify the factors influencing changes in DRD or depressive symptoms, at a 3-year follow-up point, in Malaysian adults with T2DM who received regular primary diabetes care. Baseline data included age, sex, ethnicity, marital status, educational level, employment status, health-related quality of life (WHOQOL-BREF), insulin use, diabetes-related complications and HbA1c. DRD was assessed both at baseline and after 3 years using a 17-item Diabetes Distress Scale (DDS-17), while depressive symptoms were assessed using the Patient Health Questionnaire (PHQ-9). Linear mixed models were used to examine the relationship between baseline variables and change scores in DDS-17 and PHQ-9. Almost half (336) of 700 participants completed both measurements. At follow-up, their mean (SD) age and diabetes duration were 60.6 (10.1) years and 9.8 (5.9) years, respectively, and 54.8% were women. More symptoms of depression at baseline was the only significant and independent predictor of improved DRD at 3 years (adjusted β = −0.06, *p* = 0.002). Similarly, worse DRD at baseline was the only significant and independent predictor of fewer depressive symptoms 3 years later (adjusted β = −0.98, *p* = 0.005). Thus, more “negative feelings” at baseline could be a manifestation of initial coping behaviors or a facilitator of a better psychological coaching by physicians or nurses that might be beneficial in the long term. We therefore conclude that initial negative feelings should not be seen as a necessarily adverse factor in diabetes care.

## Introduction

It is acknowledged that type 2 diabetes mellitus (T2DM) related psychological health deserves more attention (Scottish Intercollegiate Guidelines Network, 2010; Chan and Luk, 2016; Young-Hyman et al., 2016). Sources of psychological problems could arise from complex medical therapeutic regimens, worries about hypoglycemia and the complications of diabetes (Stuckey et al., 2014), unfavorable living environments and a lack of social support to adhere to medical advice (Hinder and Greenhalgh, 2012).

Consequently, a person's self-management behavior is pivotal to achieving good cardiometabolic control in adults with T2DM (Powers et al., 2015), and might also influence clinical outcomes (Dailey, 2011). As well as diabetes-specific knowledge, issues such as diabetes-related distress (DRD) and depressive symptoms (Ciechanowski et al., 2000) and their effective management (Schulman-Green et al., 2012) have been shown to affect self-management and quality of life in adults with T2DM (Thorpe et al., 2013; Fisher et al., 2014). DRD is defined as a patient's concern about disease management, support, emotional burden and access to care (Polonsky et al., 2005); it is an important condition and is distinct from depression (Fisher et al., 2014). The concept of DRD attempts to capture the emotional experiences of people with diabetes mellitus, and is content and context-specific to living with diabetes mellitus. Previous trials showed that DRD has a higher prevalence and incidence than major depressive disorders (Fisher et al., 2007, 2008). Earlier confusion of DRD and depression has now been addressed and it was proposed that “emotional distress” as a term that could cover both depression and DRD (Fisher et al., 2014). It is likely that DRD is at the milder end (but specific to diabetes) and depression is at the more severe but general end of the spectrum of mental health problems (Das-Munshi et al., 2007; Fisher et al., 2007). Additionally, both DRD and depressive symptoms are not static concepts and are likely to change over time. In Europeans with T2DM, DRD was associated with the duration of diabetes and this association could be largely explained by the presence of diabetes-related microvascular complications and insulin treatment (Kasteleyn et al., 2015).

Major depressive disorder was a significant and independent predictor of severe DRD (mean DDS-17 ≥ 3) at 18 months follow-up in people with T2DM in a study from the United States. Other predictors included female gender, experiencing more life or chronic stress, a high number of diabetes-related complications, as well as poor diet or a low exercise level (Fisher et al., 2009). In another study, depressive symptoms in people with T2DM were not significantly associated with DRD at 6 months (Ehrmann et al., 2015). However, if depressive symptoms were present at baseline, the risk of persistent DRD was almost 6-fold higher. Conversely, elevated DRD at baseline led to a 2.6-fold increase in the incidence of depressive symptoms 6 months later. In addition, female gender was a significant risk factor for persistent DRD. Younger age, a higher BMI, a higher number of physical comorbidities, female gender and not being married were significantly related to higher levels of DRD and depressive symptoms after 1 year. The same factors, with the exception of female gender, were also significantly associated with DRD at 2-years follow-up (Burns et al., 2015).

The above-mentioned studies generally showed the expected positive associations between DRD and depressive symptoms longitudinally. However, whether a *change* in distress or depressive symptoms is a more or less natural process or depends on disease and therapy-related factors is not well understood. A better understanding of which baseline factors are related to changes in DRD and depressive symptoms over time could facilitate effective psychological interventions (Gallo et al., 2005; Fisher et al., 2009, 2014; Thorpe et al., 2013; Ducat et al., 2014).

This study aimed to identify factors, in Malaysian adults with T2DM in primary care, that relate to changes in DRD and depressive symptoms after 3 years of follow-up. The impact of baseline demographics, health-related quality of life, and medical characteristics (insulin therapy, diabetes-related complications and HbA1c) on DRD and depressive symptoms were explored.

## Materials and methods

This is a cohort study that included baseline and follow-up data from a previous study, together with recent 3-year follow-up data (Chew et al., 2016b). During this period of follow-up, patients received standard diabetes care and clinical services at the respective health clinics. The study was approved by the Medical Research Ethics Committee (MREC), Ministry of Health Malaysia.

### Setting and participants

All people who participated in the earlier study (Chew et al., 2016b) and who returned for a follow-up visit to one of three public health clinics in Malaysia were re-invited. Patients who expressed suicidal thoughts or showed disturbed emotions at baseline or during the follow-up period were referred to the clinic's doctor for immediate assessment. Some were included in the follow-up cohort, whereas others were not, depending on whether they returned for the diabetes follow-up visit. Participants belonging to the cohorts were identified by an orange label on their follow-up cards and on their medical records. These health clinics were chosen because they serve different sections of the local population. One health clinic is urban and is visited mainly by patients of Chinese descent, the second is a rural clinic visited by proportionally more patients of Indian descent than found in a usual public health clinic, and the third clinic is in a rural and predominantly ethnic Malay residential area.

Participants were sampled consecutively as they came to the clinics over a period of 6 months in 2016. Inclusion criteria at baseline: age 30 years or older, a diagnosis of T2DM more than 1 year ago, and with at least three clinic visits in the previous year. The baseline exclusion criteria were pregnancy or lactating, psychiatric/psychological disorders that could impair judgment and memory, and participants who could not read or understand English, Malay or Mandarin (Chew et al., 2016b). Before the questionnaires were filled in, all participants gave written consent in their preferred language while waiting for a medical consultation with the clinic's doctor. Trained research assistants interviewed participants who were not able to self-administer the questionnaires.

### Data collection

Baseline demographic data including age, gender, ethnicity, religion, educational level, employment status and monthly income were included in the questionnaires. Structured case record forms were used for data collection from the medical records and included the duration of diabetes, HbA1c, diabetes-related complications, blood pressure, lipids, number and type of medication use (Chew et al., 2016b).

Data on hypertension (systolic blood pressure ≥ 130 mm Hg or diastolic blood pressure ≥ 80 mm Hg), dyslipidemia (LDL-cholesterol > 2.6 mmol/L or triglyceride > 1.7 mmol/L or HDL-cholesterol < 1.1 mmol/L, as well as diabetes-related complications (retinopathy, nephropathy and diabetic foot problems; or ischemic heart disease and cerebrovascular disease or stroke) were retrieved from the medical records (Chew et al., 2016b).

DRD and depressive symptoms were measured using the 17-item Diabetes Distress Scale (DDS-17) (Polonsky et al., 2005; Ting et al., 2011; Chew et al., 2015c) and the Patient Health Questionnaire (PHQ-9) (Kroenke et al., 2001; Sherina et al., 2012; Yu et al., 2012), respectively. Former studies in Malaysia supported the use of these questionnaires in Malay, English and Chinese (Chew et al., 2015a,b,c, 2016b). DDS-17 yields a total scale score plus four sub-scale scores: emotional burden, physician distress, regimen distress and interpersonal distress, with mean scores ranging between 1 and 6, with higher scores representing more DRD (Polonsky et al., 2005). PHQ-9 has a score range of 0–27, with higher scores denoting worse depressive symptoms (Kroenke et al., 2001; Sherina et al., 2012; Yu et al., 2012). The quality of life at baseline was measured with the World Health Organization Quality of Life- Brief (WHOQOL-BREF) (Yao et al., 2002; Hasanah et al., 2003; Skevington et al., 2004). DRD and depressive symptoms were measured again at follow-up but without use of the WHOQOL-BREF. All questionnaires used were locally validated and were prepared in three languages: English, Malay and Mandarin.

### Data analysis

Data analyses were carried out using PASW 21.0 (SPSS, Chicago, IL). Patient characteristics are presented as mean (SD) or median (IQR) for continuous variables, and counts and percentages for nominal variables using descriptive statistics. Comparisons of mean levels were performed using the Student's *t*-test or Mann-Whitney U test for unpaired samples, and the Chi-square test was used for proportionate samples between the participants and non-participants. The determinant-outcome relations for each factor were analyzed with linear mixed models, with a change in DRD and a change in depressive symptoms after 3 years follow-up as the dependent variables. The change scores were calculated by subtracting the scores in 2016 from the scores in 2013. A positive change score indicates an increase in DRD or depressive symptoms, while a negative change score denotes a reduction in emotional distress. These change scores capture changes at a personal level. Obviously, higher distress at baseline makes it more likely that this distress decreases over time (via regression to the mean, or via intervention). This decrease could then be accompanied by a decrease in depressive symptoms. Nevertheless, in using change scores we did not adjust for baseline scores since this might have caused biased parameter estimates (Glymour et al., 2005).

The linear mixed effect modeling incorporated the effect of time expressed as the intercept. We chose to analyze change scores because there were only two-time points in this study and the follow-up time was nearly identical for each subject. A random intercept per healthcare center was used to account for clustering. Fixed effects were used for the above-mentioned baseline patient and disease-related characteristics. These covariates were chosen based on past studies (age, gender, ethnicity, quality of life and insulin therapy) (Chew et al., 2015b, 2016a) and the baseline cross-sectional study in 2013 (marital status, educational levels, employment status, diabetes-related complications) (Chew et al., 2016b). Statistical significance was set at *p* < 0.05.

## Results

### Cohort characteristics

In total, 336 participants completed the follow-up measurements, representing nearly half of the baseline sample (*n* = 700) (Table 1). Their mean (SD) age and diabetes duration were 60.6 (10.1) and 6.8 (5.8) years, respectively. Men and women were nearly equally represented (54.8% women). The majority of the participants were Malay, were married, and educated at least up to secondary school level (Table 1).

**Table 1:** Baseline characteristics of the participants and non-participants in percentages, unless otherwise stated.

**Baseline characteristics**		**Participants *n* = 336**	**Non-participants *n* = 364**	***p-*value**
Seri Kembangan/Dengkil/Salak (% of cohort)		44.9/25.9/29.2	20.1/9.9/70.1	<0.001
Age in year, mean (SD)		60.6 (10.14)	59.3 (10.20)	0.106
Diabetes duration in year, mean (SD)		6.8 (5.78)	6.2 (5.59)	0.160
Gender	Female	184 (54.8)	184 (50.5)	0.265
Ethnicity^*^	Malay	147 (43.8)	220 (60.4)	< 0.001
	Chinese	110 (32.7)	52 (14.3)	
	Indian	75 (22.3)	90 (24.7)	
	Others	4 (1.2)	2 (0.5)	
Marital status	Married/living with partner	268 (80.2)	283 (78.2)	0.642
Education level	No and Primary education	155 (46.7)	149 (41.8)	0.051
	Secondary	152 (45.8)	158 (44.3)	
	Tertiary	25 (7.5)	50 (14.0)	
Employment	Employed	148 (44.2)	167 (46.1)	0.931
	Retired	83 (24.8)	89 (24.6)	
	Unemployed/ homemaker	104 (31.2)	106 (29.3)	
HbA1c in mmol/mol and %, mean (SD)		68 (22.2) 8.4 (2.03)	68 (24.4) 8.4 (2.23)	0.160
Hypertension (*n* = 688)		264 (80.2)	275 (76.6)	0.267
BP < 130/80 mmHg		111 (33.0)	79 (21.7)	0.001
Dyslipidemia (*n* = 673)		184 (57.3)	81 (23.0)	<0.001
LDL-C ≤ 2.6 mmol/L (*n* = 566)		121 (41.2)	108 (39.7)	0.733
Oral anti-diabetic agents (*n* = 695)		310 (92.8)	324 (89.8)	0.180
Insulin therapy (*n* = 694)		125 (37.5)	146 (40.4)	<0.001
Microvascular complications		28 (8.3)	25 (6.9)	0.479
Macrovascular complications		18 (5.4)	24 (6.6)	0.527
Total WHOQOL-BREF, mean (SD)		61.1 (10.14)	62.2 (9.57)	0.144
Overall DDS-17, mean (SD)		2.3 (1.02)	2.1 (0.84)	0.023
Emotional burden, mean (SD)		2.4 (1.13)	2.4 (1.01)	0.784
Physician distress, mean (SD)		2.1 (1.21)	1.8 (1.11)	0.005
Regimen distress, mean (SD)		2.4 (1.10)	2.2 (0.98)	0.031
Interpersonal distress, mean (SD)		2.3 (1.33)	2.0 (1.08)	0.007
Total PHQ-9 score, mean (SD)		4.8 (4.47)	4.5 (4.15)	0.338

* *Others included aborigines and other ethnicities. DDS-17, the 17-item diabetes distress scale, with mean scores ranging from 1 to 6; PHQ-9, the 9-item patient health questionnaire, with total scores ranging from 0 to 27; SD, standard deviation; WHOQOL-BREF, World Health Organization Quality of Life-Brief*.

Most participants lost to follow-up “could not be reached or located” (*n* = 301), some switched to care elsewhere (*n* = 37), one was confirmed deceased and 25 declined further participation. Compared to the participants, the non-participants differed significantly in several aspects with regard to their baseline measures: they were more often treated at the rural Salak Health Clinic (70.1, 20.1% at Seri Kembangan and 9.9% at Dengkil, *p* < 0.001), a lower proportion achieved blood pressure < 130/80 mmHg (21.7% vs. 33.0%, *p* = 0.001), and had dyslipidemia (23.0% vs. 57.3%, *p* < 0.001). Insulin therapy was also more often prescribed for non-participants (40.4 vs. 37.5%, *p* < 0.001) (Table 1). At baseline, participants had a higher overall DDS-17 score compared to non-participants. Participants had higher mean scores on physician distress, regimen distress and interpersonal distress compared to the non-participants, but did not differ on emotional burden (Table 1). Participants and non-participants did not differ in total PHQ-9 scores (Table 1). Out of the six patients who had severe depressive symptoms (total PHQ-9 score ≥ 20) at baseline, five participated and one was lost to follow-up. Non-participants had a significantly lower total number of clinic visits (mean 12.6 vs. 13.9, *p* = 0.004) but a higher number of hospital referrals (mean 1.7 vs. 1.2, *p* < 0.001) and visits to other healthcare professionals (mean 1.0 vs. 0.6, *p* = 0.048).

### Follow-up results

**Figure 1:**
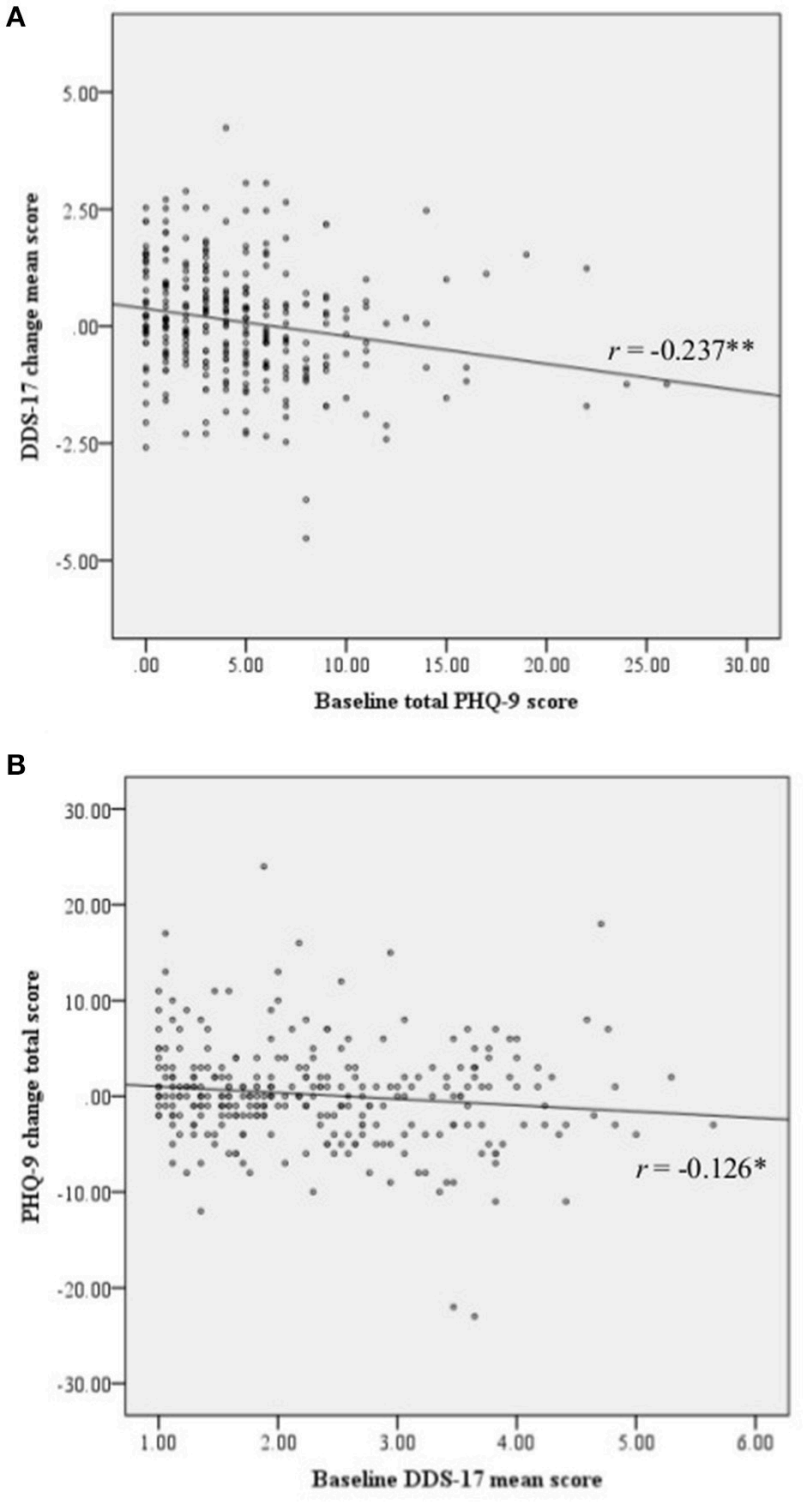
Scatterplots. **(A)** Scatterplot of baseline total PHQ-9 score and the DDS-17 change mean scores. **(B)** Scatterplot of baseline DDS-17 mean score and the PHQ-9 change total score. DDS-17, the 17-item diabetes distress scale; PHQ-9, the 9-item patient health questionnaire.

Correlations between overall baseline DDS-17 scores and PHQ-9 change scores were negative, as were correlations between baseline PHQ-9 scores and DDS-17 overall change scores (Figure 1). Table 2 shows the baseline, follow-up and change scores for the mean DDS-17 and total PHQ-9 scores of the participants. The overall DDS-17 and emotional burden subscale scores and the PHQ-9 scores increased slightly but the other scores remained the same or decreased slightly, suggesting that DRD and depressive symptoms did not change significantly over 3 years in the participating cohorts as a whole. Though the mean DDS-17 and PHQ-9 change scores were close to 0 (Table 2), there was considerable variation among participants: change scores varied from –4.53 to +4.24 and from −23.00 to +24.00, respectively.

**Table 2:** Baseline, follow-up and change scores for the DDS-17 and PHQ-9.

**Emotions**	**Mean (*****SD*****)**		
	**Baseline**	**Follow-up**	**Change in score**
Overall DDS-17	2.3 (1.02)	2.4 (0.97)	0.1 (1.23)
Emotional burden	2.4 (1.13)	2.6 (1.15)	0.3 (1.27)
Physician distress	2.1 (1.21)	2.1 (1.15)	0.01 (1.58)
Regimen distress	2.4 (1.10)	2.4 (1.07)	0.08 (1.34)
Interpersonal distress	2.3 (1.33)	2.1 (1.20)	–0.1 (1.68)
Total PHQ-9 score	4.8 (4.47)	5.0 (5.00)	0.2 (5.18)

*DDS-17, the 17-item diabetes distress scale, with mean scores ranging from 1 to 6; PHQ-9, the 9-item patient health questionnaire, with total scores ranging from 0 to 27; SD, standard deviation*.

### Predictive factors for DDS-17 and PHQ-9

After adjustment for demographic and clinical covariates in the DDS-17 change model, only PHQ-9 at baseline explained the change in DDS-17, with the higher PHQ-9 score at baseline associated with lower DDS-17 scores at follow-up. An increase of 10 points on baseline PHQ-9 was associated, on average, with a 0.6 mean score decrease in DDS-17 (Table 3). For the PHQ-9 change models, the baseline overall DDS-17 and interpersonal distress subscale scores (in two separate models) showed negative relations with PHQ-9 change scores; an increase of 1 in the mean score on the DDS-17 and the interpersonal distress subscale at baseline was associated with an average 0.98 and 0.81 improvement in PHQ-9 total score over time, respectively.

**Table 3:** Predictive factors of DDS-17 and PHQ-9 change scores, *n* = 251.

**Parameter**	**Parameter estimate**	***SE***	**95% Confidence interval**	
			**Lower bound**	**Upper bound**
DEPENDENT VARIABLE: DDS-17 CHANGE SCORE^*^				
Total PHQ-9	−0.06	0.020	−0.104	−0.023
DEPENDENT VARIABLE: PHQ-9 CHANGE SCORE^*^				
Overall DDS-17	−0.98	0.348	−1.670	−0.298
DEPENDENT VARIABLE: PHQ-9 CHANGE SCORE^*^				
Emotional burden	−0.52	0.489	−1.482	0.446
Physician distress	0.10	0.426	−0.741	0.939
Regimen distress	0.28	0.544	−0.791	1.353
Interpersonal distress	−0.81	0.410	−1.615	−0.002

* *Covariates in the model: age, gender, ethnicity, marital status, education level, employment status, insulin therapy, microvascular complications, macrovascular complications, World Health Organization Quality of Life-Brief and HbA1c. DDS-17, the 17-item diabetes distress scale; PHQ-9, the 9-item patient health questionnaire; SE, standard error*.

## Discussion

This study examined the longitudinal relationship of demographic and medical variables on the one hand and DRD or depressive symptoms on the other after 3 years of regular primary diabetes care. Unexpectedly, from all the possible baseline variables, only greater DRD and depressive symptoms were significant predictors of improved depressive symptoms and DRD at 3 years, respectively. No information was available on what occurred in the intervening 3 years. Clearly, such information would be useful in forming a more detailed picture of the determinants of changes in distress and depressive symptoms over the whole period. Nevertheless, to facilitate the development of effective psychological interventions, in this study we aimed to gain insight into which baseline factors are related to subsequent changes. This study in fact showed that there were significant converse individual changes in DRD and depressive symptoms, indicating that low DRD may predispose to greater depressive symptoms some years later, and that initially weak or absent depressive symptoms do not prevent higher DRD at a later timepoint. In our opinion, this important observation suggests that adequate psychological attention should also be offered to people with T2DM who appear to be in a relatively good psychological state.

Baseline DRD and depressive symptoms on the one hand and *change* scores at follow-up on the other showed a negative correlation, which appears to conflict with results from other studies; however, these studies examined the relations between the scores at baseline and at follow-up (Fisher et al., 2009; Burns et al., 2015; Ehrmann et al., 2015). Using the same measures for DRD and depressive symptoms in more than a thousand Canadians with T2DM, with similar demographic characteristics except for ethnicity (Burns et al., 2015), positive associations were found between baseline DRD and depressive symptoms at 2-year follow-up. However, in this study the relationship between DRD and depressive symptoms was examined directly at 1-year but only indirectly at 2-year follow-up. In that study, the coefficients at 2-years for baseline depressive symptoms predicting DRD, and for the baseline DRD predicting depressive symptoms, were 0.02 and 0.01, respectively. The negative coefficients (between the baseline PHQ-9 and DDS-17 *change* score, and between baseline DDS-17 and PHQ-9 *change* score) reported in our study were larger (0.06 and 0.98). In another study including about 500 Germans with diabetes mellitus (33% with T2DM) in an outpatient setting, higher DRD at baseline (β = 0.14, *p* = 0.001) was associated with higher depressive symptoms at 6-month follow-up (Ehrmann et al., 2015). Female gender was associated with more or more severe depressive symptoms at follow-up. Similarly, baseline depressive symptoms (β = 0.17, *p* < 0.0001) were significantly associated with DRD at 6-month follow-up. With the exception of BMI and T2DM, no other variables including gender, HbA1c, diabetes duration and late complications, were associated with DRD at follow-up. In contrast to previous studies, our study did not find any of these variables to be significant covariates (Fisher et al., 2009; Ehrmann et al., 2015). This discrepancy could be related to our use of change scores as the dependent variable, to the longer follow-up period in the present study, or perhaps more importantly, differences in socio-cultural backgrounds as discussed below.

The differing relationship between DRD and depressive symptoms in our study and the above mentioned Western studies is remarkable, especially in light of a recent study from Taiwan (Wang et al., 2016). Taiwanese adults with T2DM (*n* = 304) exhibiting higher levels of “empowerment” also had a higher level of DRD at 12-months follow-up (β = 0.154, *p* < 0.01). Additionally, increases in self-management behaviors interacted with an increase in patient empowerment and were associated with a decrease in DRD (β = –0.206, *p* < 0.01). In other words, the decrease in DRD was only observed when empowerment and self-management behaviors increased in tandem. Other covariates that were “protective” against DRD were diabetes self-efficacy and resilience. The empowerment referred to above comprised the participants' perceptions of being empowered by their healthcare providers to better illness understanding, self-awareness and personal control. Thus, it may be that our participants and those in Taiwanese both had a negative reaction to healthcare education or counseling. Likewise, well-meant support from family members or friends might have had the opposite effect to that intended (Rintala et al., 2013). However, with continuous follow-up at the health clinics and support from their “significant others” people with T2DM will subsequently improve in self-care (Gao et al., 2013) and experience less DRD and depressive symptoms. Another possible explanation for the findings in both Taiwan and Malaysia might be that certain participants expressed more DRD or depressive symptoms in order to acquire help and attention. Within the closely-knit social network typical of many Asian families and communities (Lee et al., 2013), adequate and satisfying social support was probably garnered over time, resulting in improved psychosocial well-being, less DRD and fewer depressive symptoms (Hameed et al., 2013; Karlsen and Bru, 2014). It is also possible that the respective healthcare systems offered greater support to patients who showed DRD or depressive symptoms. The negative relationship might thus indicate that patients with a low distress level and/or few depressive feelings at baseline received inadequate psychological support in the following 3 years.

To the best of our knowledge, this is the first prospective cohort study in Asian people with T2DM to examine the relation between DRD and depressive symptoms, and with the longest period of follow-up. Additionally, use of change scores as the dependent variables reduces bias in the analysis, especially when correlations between baseline and follow-up are significant (Glymour et al., 2005). However, our design also had limitations. Although similar to other studies (Silva Junior et al., 2015), the participation rate in the current study was low, suggesting some degree of self-selection. The different profiles of participants and non-participants may have had some effect on the findings of the study. The relative under-participation at 3-year follow-up of ethnic Malay participants from the rural Salak health clinics, and of those not having dyslipidemia, may have reduced the increase in the DDS-17 change score as these groups had significantly lower distress scores at baseline (Chew et al., 2016b). However, differential loss of participants does not necessarily cause bias in association estimates (Osler et al., 2008). The baseline mean DDS-17 and PHQ-9 scores in this study were 2.3 (1.02) and 4.8 (4.47), which were high compared to those of Burns et al. (2015), at 1.6 (0.68) and 4.2 (4.86), respectively. To some extent, regression to the mean may have occurred, though this statistical effect was probably limited because the mean scores for DDS-17 and PHQ-9 were higher at follow-up than at baseline. Thus, the relation between PHQ-9 at baseline and the DDS-17 change score, and between DDS-17 at baseline and PHQ-9 change score as observed in this study are plausible.

In conclusion, among Malaysian adults with T2DM who returned regularly to health clinics and received continuous healthcare treatment, initial depressive symptoms were correlated with a reduced DRD at 3 years. Similarly, initial DRD (interpersonal distress) was associated with fewer or improved depressive symptoms. These observations require a reconceptualization of DRD and depressive symptoms, at least in Malaysian people with T2DM. Evidently, such feelings and symptoms should not be considered as merely negative factors. Nonetheless, vigilant monitoring for DRD and depressive symptoms, combined with psychological support for those without emotional distress, seems a reasonable approach in diabetes care.

## Author contributions

BC, RV, and GR conceived of the study. BC contributed to every aspect of this study and drafted the manuscript. RV, RS, and GR researched the data, participated in data analysis and edited the manuscript. All authors read and approved the final manuscript, and all agreed to be accountable for all aspects of the work.

### Conflict of interest statement

The authors declare that the research was conducted in the absence of any commercial or financial relationships that could be construed as a potential conflict of interest.
